# Investigations of CNN for Medical Image Analysis for Illness Prediction

**DOI:** 10.1155/2022/7968200

**Published:** 2022-05-29

**Authors:** K. Nirmala, K. Saruladha, Kenenisa Dekeba

**Affiliations:** ^1^Department of Computer Science and Engineering, Pondicherry Technological University, Puducherri, India; ^2^Department of Food Process Engineering, College of Engineering and Technology, Wolkite University, Wolkite, Ethiopia

## Abstract

When it comes to diabetic retinopathy, exudates are the most common sign; alarms for early screening and diagnosis are suggested. The images taken by cameras and high-definition ophthalmoscopes are riddled with flaws and noise. Overcoming noise difficulties and pursuing automated/computer-aided diagnosis is always a challenge. The major objective of this approach is to obtain a better prediction rate of diabetic retinopathy analysis. The accuracy, sensitivity, specificity, and prediction rate improvement are focused on the objective view. The images are separated into relevant patches of various sizes and stacked for use as inputs to CNN, which is then trained, tested, and validated. The article presents a mathematical approach to determine the prevalence, shape in precise, color, and density in the populations among image patches to operate and discover the fact the image collection consists of symptoms of exudates and methods to comprehend the diagnosis and suggest risks of early hospital treatment. The experimental result analysis of malignant quality shows the accuracy, sensitivity, specificity, and predictive value. Here, 78% of accuracy, 78.8% of sensitivity, and 78.3% of specificity are obtained, and both positive and negative predictive values are obtained.

## 1. Introduction

Windows are open ajar for the research in DR (diabetic retinopathy) and solving problems to detect the severity of damage that occur while taking into account all vulnerabilities in the retina. The problem is also in predicting vulnerable damages early and generating early hospitalization notes for a variety of projected health and sickness conditions.

In humans, diabetes causes cardiovascular disease, renal failure, limb amputation, and vision loss. Diabetic retinopathy is an ocular sign of diabetes, commonly known as retinal coronary artery disease. Early indicators of diabetic retinopathy can be detected with retinal fundus imaging since visual problems are not obvious until diabetes worsens. A well-trained ophthalmologist can identify the disease using procedures and techniques, but there are only a few ophthalmology experts who can detect rapidly progressing diabetic retinopathy, necessitating the development of an automated diabetic retinopathy screening system to aid ophthalmologists.

According to a cohort investigation of patients in Indian hospitals, at least 15% of patients with severe disease develop diabetic retinopathy over time. In the event of a pandemic, persons with specific illnesses avoid going to hospitals and instead seek out home therapies. However, in the context of therapy and trials, the conclusive rate of succumbing to serious sickness is almost identical to the number of patients admitted to hospitals. This research work motivates to improve the image analysis with a convolution neural network and the medical imaging applications. Major contribution of this approach is to obtain a better accuracy and prediction value.

This article is summarized as follows.  Section 2 describes the review analysis of various research studies, and Section 3 presents the existing work description.  Section 4 proposes the CNN-based fundus image analysis, and Section 5 provides the experimental discussion. Finally, Section 6 concludes the proposed work with better analysis state and future work.

## 2. Literature Review

With the contributions of LeCuN in 1989, convolutional neural networks were thrust into the spotlight. This was only applied to topological data. Hubel (1962) and Wiesel (1968) were the first to inspire the CNN's design and architecture, and the basic structure continues to influence the majority of ongoing contributions. In monkeys, the visual system and ventral pathways of the visual system are mimicked by CNNs.

Image classification and segmentation are the most chosen prominent methods in image processing, which have due importance along with feature extraction. Deep convolutional neural networks, for example, provide a similar purpose but with more differentiating characteristics. The DCNN plays a very important role in the detection of exudates in the images of retinal fundus. Deep convolutional neural networks' convolution layers outperform machine learning tasks without making the same mistakes.

Tan et al. [1] built a convolutional neural network that can automatically distinguish the elements of diabetic retinopathy. Convolutional neural networks have the ability to generate spectacular breakthroughs in the processes of segmentation and classification in vast volumes of digital fundus retinal datasets with the highest levels of accuracy. Garcia et al. [2] proposed using a multilayer perceptron, to detect hard exudates in large datasets of diabetic retinopathy digital fundus images. Xiao et al. have annotated a detailed study of exudates identification in digital fundus images of diabetic retinopathy, as well as new learning techniques for classification and feature extraction.

Deep architectures enhance the advantages of shallow designs when dealing with complex learning challenges. The ability to learn complicated representations is improved by layering nonlinear and linear processing modules. The performance of deep architectures of convolutional neural networks is significantly better than that of standard vision-based models.

Although basic pathological examination and the detection of lesions are important in the diagnosis of diabetic retinopathy, the diabetic retinopathy literature generally agrees that the most common and prominent pathological sign of diabetic retinopathy is “Detection of Exudates,” which is the most common and prominent pathological sign of diabetic retinopathy. Narang et al. [3] propose to detect and classify hard exudates, an algorithmic technique including split, predict, and update, as well as the framework. They claim that simple image processing is sufficient for diagnosing diabetic retinopathy, which is a preprocessing step in the learning process. Rajput et al. [4] created mathematical morphologies that were coupled with the k-means approach and applied to the CIELAB color space and preprocessed photographs. In order to showcase the peculiarities of exudates, the cited works make a good effort to remove noise and other unnecessary components. The authors of the study performed using the e_ophtha EX and DIARETDB1 datasets found that hard exudates are early indicators of diabetic retinopathy. They also found that hard exudates are associated with increased risk of developing diabetic retinopathy. Their research develops a framework for training a model of hard exudates identification using multilayer perceptron-induced supervised learning, which is then implemented in practice. “Hard exudates segmentation based on learned initial seeds and iterative graph cut.” The hard exudates are extracted with high certainty using an iterative graph cut approach. Hard exudates were discovered by morphological operations, according to Mahdi et al. [5]. To segment the hard exudates after preprocessing the fundus image to remove the optic disk and retinal blood vessels, the researchers employed a number of morphological techniques such as the top hat, bottom hat, and reconstruction processes to segment the hard exudates. For surfacing hard exudates, with the help of color histograms, Sanchez et al. [6] developed an estimating strategy for exudates. The goal of these methods is to surface exudates while distinguishing them from their surrounding backdrops. A clustering method based on the statistical mixture model, which is also utilized in dynamic thresholding, is employed to identify exudates from the surrounding environment. Giancardo et al. [7] suggest a color and wavelet transformation strategy for feature extraction for exudates extraction, which is based on color and wavelet transformation. The works show a series of trained SVM classifiers designed for surfacing exudates, with AUCs ranging from 0.88 to 0.94 depending on the dataset. Fraz et al. [8] developed multiscale segmentation algorithms to surface exudates, combining feature extraction and crucial morphological reconstruction filters with bootstrap decision tree classification. Kaur and Mittal [9] proposed a method for recognizing hard exudates with borders that had a sensitivity of 88.85 percent in lesion-based detection and 94.62 percent in image-based detection, using dynamic thresholding.

## 3. Existing Methods

In order to determine the classes of images that pertain to diabetic retinopathy with exudates, CNN image segmentation and classification are used in conjunction with each other.

Table 1 shows the comparative analysis from the consensus of works quoted by researchers with regard to the lesion detection and fundus classification. Some of the observations from the contributors have been tabulated, which represented performance evaluation metrics on the studies of DIARETB1, Kaggle, and MESSIDOR indicating the various types of procedures.

The ZFNet [10] is based on the faster version of R-CNN called faster R-CNN and is based on the principles underlying optic disc localization with the Hessian matrix, which is produced using the Faster R-CNN. The accuracy of the studies is approximately 99.90 percent, while the sensitivity is approximately 87.00 percent. The retinal fundus images are initially categorized as having or not having an optic disc, with the CNN being able to classify whether or not an image has an optic disc. Alghamdi et al. [11] provided a method for classifying the locations. The studies have an accuracy of about 99.20 percent and a sensitivity of 89.00 percent. Xu et al. introduce a visual geometry group-based fundus classification model with 2D convolutions and max-pooling layers. To determine pixel thresholds, the probability map with the center of gravity is utilized and [12] and identify the optic disc The studies have an accuracy of about 99.40 percent and a sensitivity of 86.00 percent. A controlled CNN model is created by Abramoff et al. [13] to classify the macular edema lesion type. The studies have nearly 96.00 percent accuracy and 100.00 percent sensitivity; nevertheless, the sensitivity and specificity have an area under curve of 78.90 percent. Methods for detecting hemorrhages are proposed in [14]. In the model, a 10-layer CNN is used to extract a sized partition of the picture from the original image, which is then labeled and classified. The trials reached about 97.00 percent accuracy, with a sensitivity of 93.10 percent; nevertheless, the sensitivity and specificity have an area under curve of 97.90 percent. Nonetheless, for fundus classification and optic disc localization, transfer learning [15] was used. The images from the MESSIDOR digital fundus imaging of diabetic retinopathy datasets were appropriately graded in roughly 1748 samples. The investigations achieved an accuracy of about 96.54 percent, with a sensitivity of 87.00 percent; however, the sensitivity and specificity have an area under curve of 99.90 percent. Reference [16] proposes a novel CNN model for extracting local distinctive features from visual words concepts, as well as a corpus of visual words for search operations. The method is now known as speed-up-robust-properties (SURP/SURF) in the works. Gargeya and Leng [17] investigated to have an accuracy of 96.00 percent and a sensitivity of 80.00 percent; nevertheless, the sensitivity and specificity have a combined area under curve of 94.00 percent. In their work, Wang et al. [18] used attention and crop networks to find suspicious patch spots in the picture map during diabetic retinopathy detection. The trials had an accuracy of about 94.20 percent, with 89.30 percent sensitivity; however, the sensitivity and specificity had an area under curve of 95.70 percent. Chen et al. [19] improve on fundus categorization by employing varying kernel sizes after the pooling layer. The trials reached about 97.00 percent accuracy, with a sensitivity of 93.10 percent; nevertheless, the sensitivity and specificity have an area under curve of 97.90 percent. The location of blood vessels and the use of a procedure known as “pretreatment” for bound component analysis are also used to detect lesions. The investigations achieved an accuracy of about 91.20 percent, with an 86.00 percent sensitivity; however, the sensitivity and specificity have an area under curve of 96.50 percent. The method for identifying lesions starts with dimensionality reduction using support vector machines, followed by classification. This method was suggested by Mansour [20] et al. on Kaggle for analyzing digital fundus images of diabetic retinopathy. The trials produced about 97.90 percent accuracy and 96.20 percent sensitivity, with an area under curve of 96.20 percent for both sensitivity and specificity. In the study by Quellec et al. [21], annealed heat maps were used to categorize the fundus, and a CNN model was utilized to support and develop this method for detecting DR lesions; however, the heat maps were not optimized as part of the procedure. There is a sensitivity of 85.00 percent and an accuracy of 90.00 percent in the studies. The sensitivity and specificity have an area under the curve of 95.50 percent and 95.00 percent, respectively.

## 4. Proposed Method

### 4.1. Collection and Preprocessing of Images

For diabetic retinopathy, there are a plethora of open-access datasets. MESSIDOR [22], DIARETDB [23], IDRiD [24], and Kaggle 2015 are a few examples. Expert ophthalmologists verified and certified these datasets. Level 0 of the DIARETDB Calibration Database contains 219 retinal fundus images, 25 of which are healthy and 194 of which have diabetic retinopathy components.

DIARETDB1 [25] is the first level of calibration. Kuopio University Hospital in Finland has packaged the dataset, which includes 28 training sets and 61 testing sets. A total of 89 retinal fundus images were acquired at a FOV of 50 under somewhat diverse conditions for a total of 89 retinal fundus images. The four experts tagged the maps with various types of lesions and noted the regions where microaneurysms and hemorrhages occurred. Images from DIARETDB1 were preprocessed and fine-tuned before being used in studies. Some of the photos taken at ophthalmological facilities are raw and must be treated before they can be classified and segmented properly. Synthetic photos were also taken from real-time settings, and some of the photographs from real-time settings are synthetic.

The most crucial and fundamental purpose of the experiment is to classify and segment fundus images. Fundus images of diabetic retinopathy were obtained with the help of ophthalmologists from ImageNet-DIARETDB1 v1.1, IDRiD, and synthetic images using ImageNet-DIARETDB1 v1.1, IDRiD, and synthetic images. This collection includes images in a range of formats, resolutions, and color palettes. To meet the uniform distribution that underpins the experiment, the entire collection of candidate photos is gathered, normalized, and rescaled. The images are sorted and organized based on the patient's physiological features, including weight, age, gender, and other morbid conditions.

### 4.2. Denoising

Rescaled and categorized images are taken from the collection. The pictures are rescaled to extract different levels of detail from diabetic retinopathy features, such as abstract data, component-by-component data, and component status. The images of better resolution serve as references for diabetic retinopathy components in the initial step of the technique, which evaluates low-quality photographs to assess whether they contain diabetic retinopathy. Further photos with more severe features are used as references for photographs with a higher level of resolution, and traces of diabetic retinopathy effects are examined. Image sets with 360 × 360 resolution images that state abstract information about the images are created, as are image sets with component information about the images that are created, and image sets with status information about the components in the images that are created. Image sets with 360 × 360 resolution state only abstract information, as are image sets with 480 × 480 resolution images that state component information.

Noise filters are applied to the photos that have been captured. Noise in an image is a random variation of intensities that appears as grains in the image. This is most likely caused by low-resolution photos, inadequate lighting, or thermal energy from the image sensors. The visual quality suffers from noise. Because the photographs are transmitted through the Internet, there is a chance that they will contain inaccuracies.

When noise filters are applied to an image, the noise is removed. When working with low-resolution photos, a smoothing filter is applied initially, followed by a median filter, which is applied after the image has been sharpened to overcome the object complexity in the image.

Using the weighted sum, of pixels, a linear filter is constructed in each of the successive windows. Because the linear filter is spatially invariant, a common weight pattern is employed in subsequent windows. For a 3 × 3 smoothing filter, a typical weight pattern is mentioned in Table 2.

Linear smoothing filters are used to reduce high-frequency components, resulting in the loss of sharp details.

A median filter is a nonlinear filter that selects a window of pixels and determines each window's noisy pixel. A stratified window is applied to the image in order to locate and remove the noisy pixels. This algorithm is fed significant pixel values in order to keep the pixels relevant to the object in the image and discard the rest. The median filter is useful for dealing with various types of noise in photos; however, it is less commonly employed because it tends to reduce image details, while lowering noise, as the stratified window's pixel values will be averaged. However, because the color palette of diabetic retinopathy fundus images is limited, the media filter was determined to be more important and easy to adopt. The image is only processed in one color because the exudates' object color is yellow. In the image, the remaining pixels are removed. The median filter is used to correct areas with noisy pixels in the exudates.

### 4.3. Exudate Detection

A gradient analysis procedure is then applied to the preprocessed images. With a given color gradient, the gradient analysis process will use edge detection mechanisms. The gradient of the objects in diabetic retinopathy fundus images is nonuniform, especially near the borders of the exudates.

**Figure 1:**
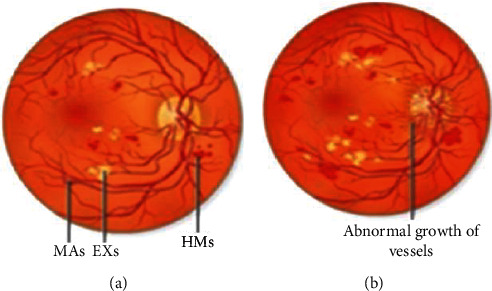
Symptoms of exudates in digital fundus images of diabetic retinopathy.

Various types of exudates: Diabetic individuals are at risk for vision loss, which can be recognized with regular checkups and corrected if caught early enough. The retinal fundus pictures of such candidates are collected and evaluated for the presence of watery substances in the retina. Vision loss is caused by the accumulation of watery substances in the blood vessels; dense accumulations of watery substances are yellow in color and are referred to as hard exudates; thin accumulations of watery substances are white to pale yellow in color and are referred to as soft exudates. Exudates in diabetic retinopathy fundus pictures take on a variety of forms. A two-variable function is used to calculate a gradient trace in general. Identifying the gradient requires locating the places with the greatest possible intensity increase and rate of change of direction.  Figure 1(a) depicts the negative impact of multiple diabetic retinopathy lesions, including microaneurysms, exudates, and hemorrhages.  Figure 1(b) shows aberrant vascular growth in the retina as a result of unfavorable components. Exudates are indicated by a yellowish-white deposit on the peri circumference of the diseased artery. Exudates are easily visible in comparison to other symptoms.

Some parts of the retina are covered in a strange yellow tint known as hard exudates, which is commonly mistaken for the optic disc. Soft exudates contain a significant amount of blue color; nevertheless, hard exudates are pale yellow in color, the exudates regions admixing despite the fact that soft exudates are frequently whitish yellow in color, making the separation of hard exudates problematic. The precise distinction of hues can be aided by the use of appropriate lighting conditions, and separation may be simple. Soft exudates can have a little bit of blue tinge.

### 4.4. Irregular Shapes of Exudates

In order to detect gradients, the method takes into account the widths of canny edges as well as the distance of coverage of higher intensities during the detection process. Elliptical annotations are used to describe the edges of the regions recognized as exudates in diabetic retinopathy fundus images that show up as nonuniform enclosed shapes. Exudates are represented by the covered gradients, which are the edges of the regions, generally in the nonuniform enclosed shapes. The axes of an ellipse are responsible for determining its shape.

**Figure 2:**
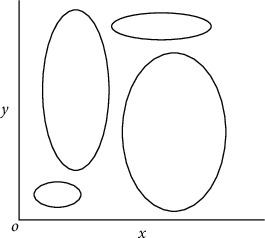
Probable shapes of elliptical objects in the imaginary Cartesian plane.

In Figure 2, after being compared to the candidate's selected major and minor elliptical axes, it is concluded that the annotated elliptical regions are potentially detrimental to his or her failing health. Using all of the samples of diabetic retinopathy fundus images together, the sets of annotated elliptical regions can be used to predict the candidate's future health concerns. Using Find-Max-Elliptical-Zone, the maximal elliptical region in diabetic retinopathy fundus images is identified and compared to the severity threshold size, suggesting that the candidate has a medical emergency. When a viewer views a retinal fundus image, this method is offered to alert them to the presence of a larger ellipse within a set of elliptical regions. The process of obtaining the maximum elliptical region is applied to the candidate retinal fundus image, and the areas of the elliptical regions are mathematically compared to the threshold area in order to determine the best candidate.

Area of an ellipse ⇒ (*x*^2^/*a*^2^)+(*y*^2^/*b*^2^)=1; for ***a*** > ***b***.

#### 4.4.1. Find-Max-Elliptical-Region

#### 4.4.2. Hue Equalization and Normalization Method

Brightness, contrast, and colors of the benchmark and synthetic images varied because they were taken under different lighting conditions. The gross-average color will be stabilized in order to continue the investigation forward. Hue is defined as the ratio of green to red pixel color values, adjusted to the hue's average pixel value. Consider the image *X*, where the RGB components are *X*_r_, *X*_g_, and *X*_b_, and a pixel at (***i***, ***j***) containing *X*_r_(***i***, ***j***), *X*_g_(***i***, ***j***), and *X*_b_(***i***, ***j***).

Therefore, [*H*(*i*, *j*)]=[*X*_*g*_(*i*, *j*)/*X*_*r*_(*i*, *j*)] for all ***X***_***r***_ (*i*, j) > r_**T**_ > 0.

For the sake of avoiding division by zero in the preceding equation, the value of *X*_r_(***i***, ***j***) is maintained by the use of the threshold rT, where the value of rT is always a very small positive value of 0.1.

If H_***des***_ is the desired average hue, which is typically 0.5160 and generated from the image's intensities, an iterative point-by-point procedure of adjustment/correction () is carried out with the participation of red and green intensities, culminating in the development of the hue matrix Hc I j):

(1) $$\begin{array}{c} \left[H_{c}\left(i,j\right)\right]=\left[\frac{X_{g}\left(i,j\right)}{X_{r}\left(i,j\right)}\right]. \end{array}$$

It is further compared with Hdes, and the value for adjustment/correction I j) is identically made comparable to Hc average I j). The average of Hc is indicated as Hc average I j).

This implies ***H***_***c_average***_ ≈ ***H***_***des***_

### 4.5. Histogram Methods

Diabetic retinopathy digital fundus shots are typically not monotone photographs with dark backgrounds, but rather deep hues that are not visible in regular light. The image components discussed in the previous sections are used to classify diabetic retinopathy digital fundus images. The first indicators of diabetic retinopathy are exudates, which indicate the extent of the proliferative process. Exudates come in a variety of colors, including pale yellow, yellow, and bright yellow.

At the preprocessing stage, the standard color-based image retrieval methods are used. RGB palettes are used to represent the colors in the retinal fundus picture. 16581375 is the total number of colors calculated. The use of histogram analysis to summarize the intensity and distribution of colors in photographs is beneficial. In order to represent each component's bit value in the histogram with as few and distinct a range of pixel values as possible, the fundus pictures are represented by 48 bins, each of which contains a potentially discrete range of pixel values. In the image component, each counter bin can be computed. for each range of pixels that occurs in the image. Normalization is performed to the ratio of the pixel in bin number with number of pixels present in the image.

Gray-level co-occurrence matrix, formerly known as gray-level coherence matrix, is one of the most basic foundational approaches in histogram equalization. They are the only gray-scale imaging systems that work, despite their simplicity. The GLCM is used in this study to look into the detailed evolution of the topic of picture component identification in the region of interest. A basic analysis of the GLCM utilizing the black-yellow spectrum indicated certain limitations in recognizing the image component in the region of interest because to the great contrast. As a result, adaptive histogram equalization should be used in the experiment. High levels of albedo, which are prevalent in photographs taken in bright light, are a general obstacle to this procedure. Because light passes through the retinal fundus images to establish depth, large level contrasts in the image components' border areas are a possibility. To further personalize the digital fundus images of diabetic retinopathy, a contrast limited adaptive histogram equalization with RGB density is applied in conjunction with RGB density. This allows us to determine the pace at which image components from the regions of interest are acquired. Before creating a histogram for the region, it is contrasted in order to eliminate any additional noise from it. A sampled image in the region of interest is used to generate several histograms, which are then displayed. It is believed that dense pixels with values that are equivalent to the yellow spectrums indicate macular retinopathy, and their values are signaled in the GLCM by color ranges that are connected with them. Furthermore, the existence or absence of exudates is determined by the symptoms.

As a result of the exercises, a Contrast Limited Adaptive Histogram Equalization (CLAHE) with RGB saturations is developed, a fresh and simpler approach that may be applied to biomedical images in particular. Rather than examining the entire image from the source to see whether exudates are there, this approach detects whether or not a fundus image has exudates. Other photographs are considered noisy or do not exhibit exudates; thus, the experimentation accuracy is up to 98.67 percent. After repeated resamplings, the average percentage of correctness remained at 97.6 percent (at least 50).

Table 3 illustrates the undesirable health issues depending on the diameters of exudates is specified.

#### 4.5.1. Network Setup and the Parametric ReLU (*p*ReLU)

The CNN is introduced with a sequential model consisting of two convolutional layers: ReLU and (pReLU) parametric ReLU, with pReLU parameters obtained by computing the geometric mean of two positive values.

The ReLU employs the geometric mean to exponentially increase the model's learning behavior. The activation function ReLU (rectified linear unit) is described technically as *y* = max (0, *x*). The model with ReLU is less expensive and faster because it requires less arithmetic. In Table 4 for all positive values, ReLU is linear (identity), whereas for all negative values, it is zero. However, in the situation of sparse data, ReLU suffers from the majority of zero values, resulting in a null activation function and a failed learning model. Negative values in leaky ReLU are supposed to have a tiny slope of 0.01 or 0.001, and some slope is created using parameters from the outside world instead of assuming a tiny slope value.

Geometric is a term we use in our work. If the next value is zero, the mean of the last two positive values is computed as the setup. As a result, a proper interpolation is done, and the model's learning is profitable. CNN architecture is used to find feature exudates. Feature exudates reveal the peculiarities of a candidate's health problems. A CNN architecture that can classify diabetic retinopathy fundus images with feature exudates for categorization. In the picture data domain, general CNN models are widely used. Image categorization, object detection, and image recognition are all well-known techniques. The proposed convolutional neural network contains four convolution layers, each with 16 feature maps. The ReLU is used to avoid saturations. Kernel size is 2 × 2 each max pooling layer, and normalization layers are used to faster convergence. The max-pool layer yielded sixteen features, which were then supplied to the 256-neuron fully connected layer. At the last stage, four target classes obtain the output.

In Table 5, segments are developed to the basis of specific pathological symptoms with a sample size of image patches of 50 with segment size *S*. The relevant patches, on the other hand, are manually extracted from 75 DIARETB1 retinal fundus photos and employed in the training process instead. A total of 23,326 patches were found to have pathological symptoms of exudates, while 52,336 were found to be negative. All of the patches have been determined to be nonoverlapping.

Model accuracy and mistakes are recorded using the validation set after the model has been run for any number of epochs ranging from 0 to 100. Because the accuracy reaches saturation at the 49th epoch, the maximum number of training epochs is taken into consideration (96 percent). A statistical summary in the above table is provided indicating the number of patches examined for the CNN's training (75 percent), validation (15 percent), and testing (15 percent) phases.

## 5. Experimental Results

For a total of ten repetitions, the experiment is repeated. In the experiment, a patch-based evaluation method was used. In Table 6 evaluation metrics for the iterations of the experiment are sensitivity, specificity, and accuracy, which designate the outcomes as the best for exudate diagnosis. The accuracy, sensitivity, and specificity for diagnosing the existence of pathological symptoms of exudates were 0.978, 0.962, and 0.979, respectively.

The multiple categories of predictions are projected in Table 7, which are drawn from the experiment for the image patches supplied as input.

**Figure 3:**
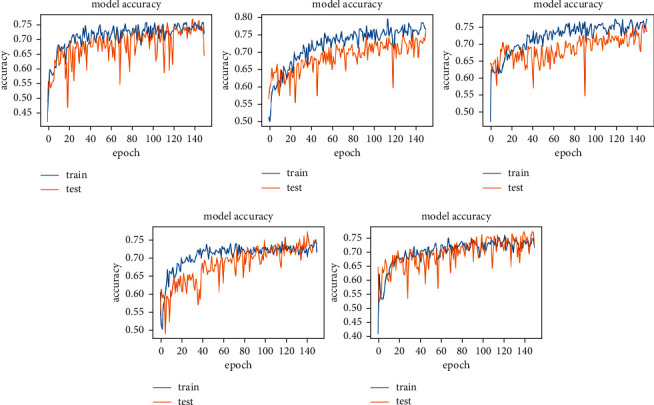
Assertion of the model accuracy in DCNN (detecting the symptoms of hard exudates) with epoch graphs generated in Keras.

The experiment is conducted on DIARETB1, a benchmark dataset, as well as on some synthetically generated datasets. According to the observations, as the number of repeats increases, the mean squared error reduces, converging the loss value for learning as close to zero as possible. The suggested DCNN model's learning performance is depicted in a graphical manner utilizing a repeater operating characteristic curve shown in Figure 3.

**Figure 4:**
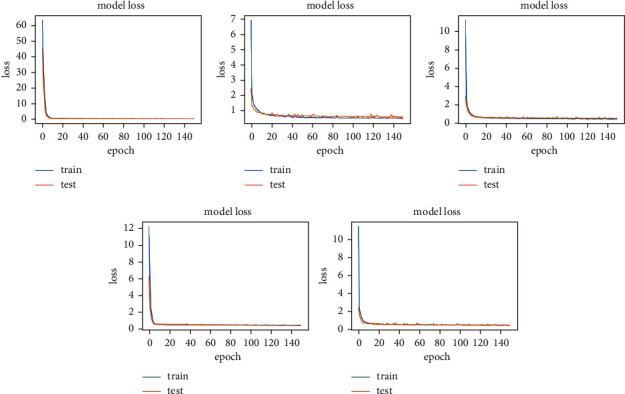
Assertion of the loss in the model in DCNN (detecting the symptoms of hard exudates) with epoch graphs generated in Keras.

Keras is also used to run the experiment on the DCNN's “sequential” model. The observatory's observations are graphically represented here. In each epoch of the experiment, there are 250 samples, and the network is trained and tested across a total of 150 epochs of the experiment shown in Figure 4.

**Figure 5:**
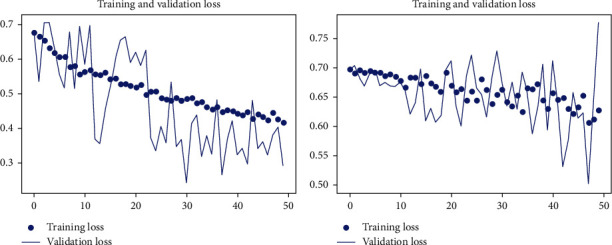
Training and validation loss due to misclassification of images, or images very far from the criterion of the classifiers.

**Figure 6:**
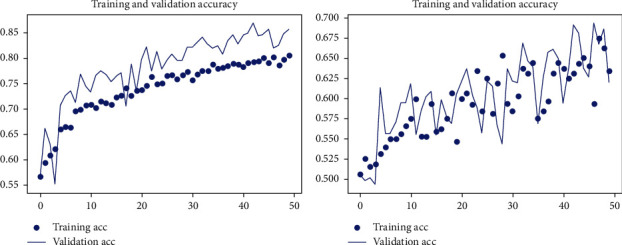
Training and validation efficiency of images, where images contain all the features mentioned by classifiers.

Figures 5 and 6 show training and validation of images submitted to the proposed network, which were completed with minimum loss and good accuracy thanks to the suggested network.

A potential conflict of interest could lead to picture grading mistakes, which could damage the model's generalization capacity. The ROC curves for soft and hard exudates were created (Table 8).

**Figure 7:**
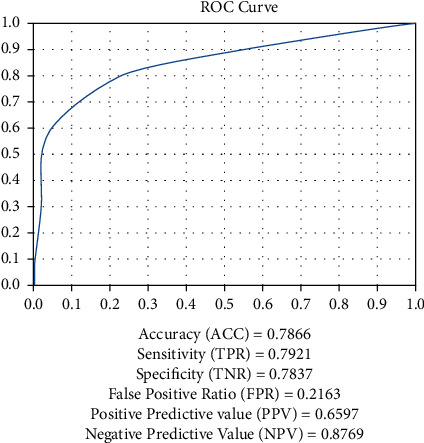
AUC describing the efficiency of the classification of digital fundus images of diabetic retinopathy that were examined for the symptoms of exudates.

The images in the table above represent the observable and cumulative collections of diabetic retinopathy images that were investigated for the features of exudates in Table 9. In Figure 7 the ROC curve for the above data is displayed, and the area under the curve (AUC) was 0.889515, indicating around 88 percent of the image characteristics of exudates. Based on the benign quality-based funds image analysis, the result is given as accuracy is 78.6%, sensitivity is 79%, and specificity is 78.3%.

**Figure 8:**
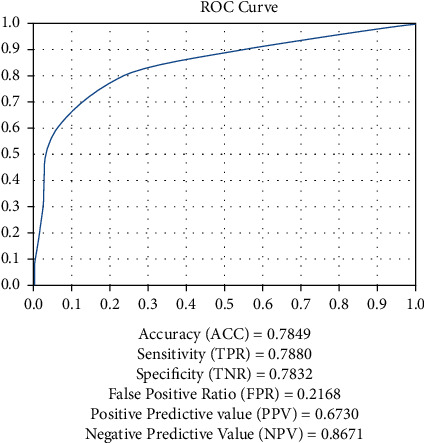
AUC of the ROC curve describing the efficiency of the classification of digital fundus images of diabetic retinopathy that were examined for the least count of the symptoms of exudates.

The digital fundus images of diabetic retinopathy in the table above were examined for malignant soft and hard exudate characteristics, as well as for observable and cumulative collections of digital fundus photos. According to Figure 8,, the AUC of the ROC curve for the above data was 0.882299, implying that over 88 percent of the photos had malignancy characteristics.

The experimental result analysis of malignant quality shows the accuracy, sensitivity, specificity, and predictive value. Here, 78% of accuracy, 78.8% of sensitivity, and 78.3% of specificity are obtained and the both positive and negative prediction values are obtained.The scaled images with various dimensions of diabetic retinopathy for the detection of features related to exudates, the model is considered to have beautiful metric performance, according to the researchers. The general purpose of the experiment is to find the cancerous images among all of the diabetic retinopathy digital fundus photos collected at various resolutions. The suggested CNN model is meant to accept 240 × 240 pixel cancerous images, as well as convolution and pooling layers with activations in pReLU with GM, as inputs. Modeling and simulation are carried out in Python, utilizing Tensorflow, Keras in Anaconda Navigator 3, and Jupyter Notebook. For the purpose of demonstrating the utility of the proposed paradigm, the experiment is carried out on a Google Colab with a single 12 GB NVIDIA Tesla K80 GPU.

**Figure 9:**
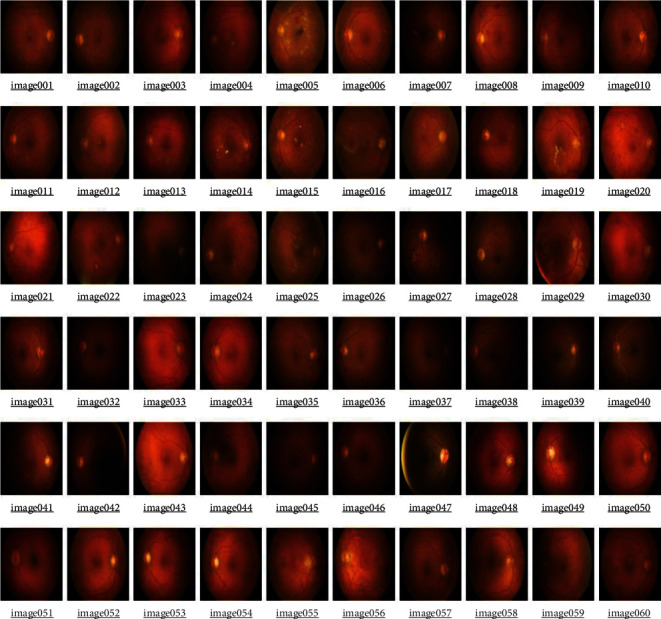
Images from DIARETB-Calibration 1—selected for generating patches—while identifying potential images with symptomatic patches of exudates.

Macular retinopathy is defined as images with a density of pixels in the RGB(255,178,102) to RGB(255,128,0) color range, that is, #ffcc99 to #ff8800. These symptoms also define the presence of exudates shown in Figure 9.  Table 10 shows comprehensive view of classification and preprocessing in a DCNN with proposed GMPR-PReLU along with the efficacies of GLCM, AHE, and CLAHERD in the proposed DCNN.

## 6. Conclusions

By training the network with a variety of image sizes and phasing out a large number of extraneous regions of the images, a standard sequential model of deep CNN with four layers is developed to ease the classification of digital fundus images of diabetic retinopathy in humans. The network has been trained to recognize the general structures and textural properties of exudates images at various resolutions and layers. There is a medical emergency if the size of the elliptical sections that covered the exudates is bigger than the retina's minimal area. The CNN-based framework was used to assess retinal fundus pictures for pathological indications indicating exudates in this investigation. On the three distinct resolution levels, the rescaled photos were preprocessed by applying noise filters. The presence of exudate is determined by elliptical annotations on rescaled images, which are raised in contrast and divided into patches. These photos are used by CNN to train and test its employees. The network determines whether a pixel belongs to a class of pathogenic component of exudate or background. The severity of diabetic retinopathy will be determined by the chance of exudates present. The trials are carried out on the “Google Colab” platform, which employs graphics processing units (GPUs). Two publicly available standard datasets were used for experimentation in the performance evaluation. As a result, we conclude that pathologists working in the field of diabetic retinopathy can simply implement this model with less financial interference. In the future, the work may be extended with the various analysis of deep learning techniques and improvement in the feature analysis.

## Figures and Tables

**Algorithm 1:**
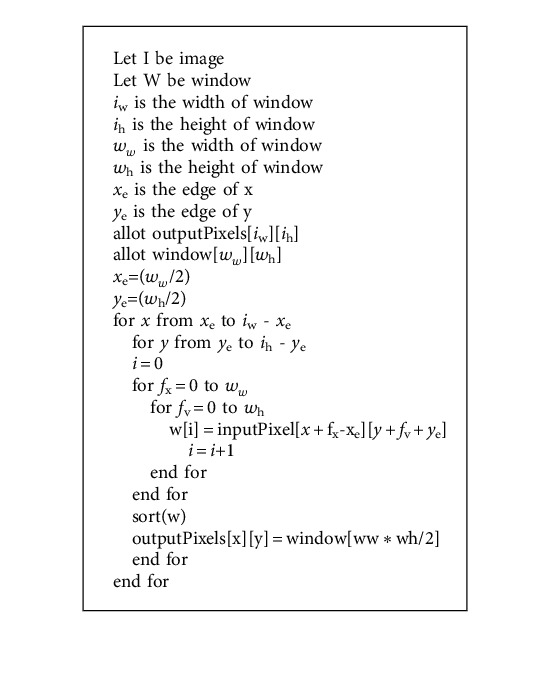
Median filter algorithm on an image.

**Algorithm 2:**
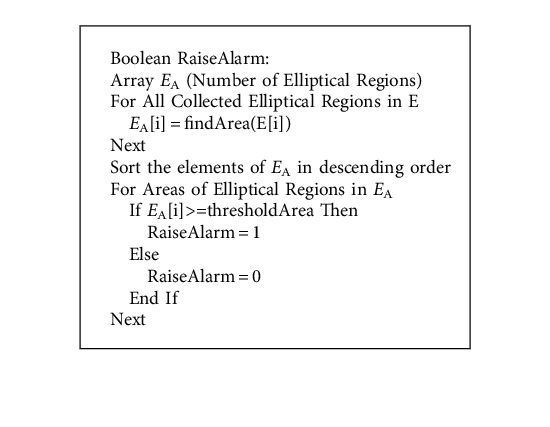
A pseudocode to find max sized ellipse in the Cartesian plane.

**Algorithm 3:**
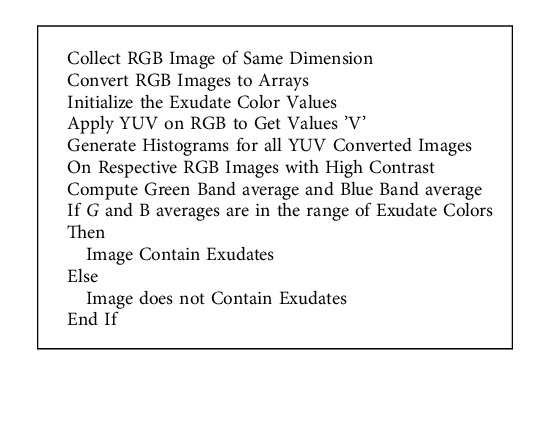
A pseudocode to apply color to select the exudates from image patches.

**Table 1:** Representation of performance evaluation metrics and repeater operating characteristic properties of the existing works.

Authors	Accuracy	AUC	Sensitivity	Process
Zhang et al. [10]	99.90	—	87.00	OD
Alghamdi et al. [11]	99.20	—	89.00	OD
Xu et al. [12]	99.40	—	86.00	OD
Abramoff et al. [13]	96.00	78.90	100.00	LD
Van Grinsven et al. [14]	97.00	97.90	93.10	LD
Gulshan et al. [15]	96.54	99.00	87.00	FC
Costta and Campilho [16]	98.30	90.00	89.00	FC
Gargeya and leng [17]	96.00	94.00	80.00	FC
Wang et al. [18]	94.20	95.70	89.30	FC
Chen et al. [19]	91.20	96.50	86.00	FC
Mansour [20]	97.90	96.20	96.20	LD
Quelle et al. [21]	92.00	95.50	84.00	FC

**Table 2:** Pattern of weights used in filters.

1/16	1/8	1/16
1/8	1/4	1/8
1/16	1/8	1/8

**Table 3:** Identification of image patches using the reference values of *R*, *G*, *B* of the color contrast ratio.

Contrast ratio	RGB			No. of fundus images	No. of image patch samples
	*R*	*G*	*B*		
	255	128–178	0–102	Total: 89	Average size: 10
16	255	128	0	30	12
18	255	136	14	40	8
20	255	144	28	50	10
21	255	152	42	60	12
22	255	160	58	65	15
24	255	166	72	65	8
26	255	172	86	70	8
28	255	178	102	75	12

**Table 4:** Validation parameters.

Parameter	Computation
Accuracy	(TP + TN)/(TP + TN + FP + FN)
Error rate	(FP + FN)/(TP + TN + FP + FN)
Positive predict value (PPV)	TP/(TP + FP)
Sensitivity	TP/(TP + FN)
Specificity	TN/(TN + FP)

**Table 5:** Setup of image patches for the experiment.

No. of images (patches) with exudates	
Training	17495
Testing	3500
Validation	3350
Total number	23326

**Table 6:** Parameters to validate the experiment.

	Exudate	No-sign
Accuracy	0.978	0.956
Sensitivity	0.962	0948
Specificity	0.979	0.966
PPV	0.939	0.958

**Table 7:** Validations of number of images per class—as obtained from the experiment.

Positive class		Negative class	
Positive prediction	Negative prediction	Positive prediction	Negative prediction
True positive	False negative	True positive	False negative
46	6	43	38
52	7	37	33
55	7	34	30
62	8	27	24
68	9	21	19
74	9	15	14
76	10	13	12
80	10	9	8

**Table 8:** Using digital fundus images of diabetic retinopathy, the DCNN can determine whether or not there are any benign qualities in the soft and hard exudates.

Benignity	Observed		Cumulative		FPR	TPR	AUC
	True	False	True	False			
			0	0	1.000000	1.000000	0.064516
1	34	3	34	3	0.935484	0.989247	0.118259
2	63	7	97	10	0.815939	0.964158	0.160998
3	88	11	185	21	0.648956	0.924731	0.184244
4	105	14	290	35	0.449715	0.874552	0.204117
5	123	23	413	58	0.216319	0.792115	0.142791
6	95	60	508	118	0.036053	0.577061	0.009855
7	9	75	517	193	0.018975	0.308244	0.003509
8	6	41	523	234	0.007590	0.161290	0.001224
9	4	30	527	264	0.000000	0.053763	0.000000
10	0	15	527	279	0.000000	0.000000	0.000000
	527	279					0.889515

**Table 9:** Using digital fundus images of diabetic retinopathy, the DCNN can determine whether or not there are any malign qualities in the soft and hard exudates.

Observed		Cumulative	
Malignity	True	False	True	False	FPR	TPR	AUC
			0	0	1.000000	1.000000	0.082437
1	46	6	46	6	0.917563	0.981013	0.126582
2	72	8	118	14	0.788530	0.955696	0.157570
3	92	12	210	26	0.623656	0.917722	0.177624
4	108	15	318	41	0.430108	0.870253	0.185592
5	119	26	437	67	0.216846	0.787975	0.132741
6	94	68	531	135	0.048387	0.572785	0.013344
7	13	78	544	213	0.025090	0.325949	0.004673
8	8	52	552	265	0.010753	0.161392	0.001735
9	6	32	558	297	0.000000	0.060127	0.000000
10	0	19	558	316	0.000000	0.000000	0.000000
	558	316					0.882299

**Table 10:** A comprehensive view of classification and preprocessing in a DCNN with the proposed GMPR-PReLU along with the efficacies of GLCM, AHE, and CLAHERD in the proposed DCNN.

GLCM with GMPR-PReLU	AHE with GMPR-PReLU	CLAHERD with GMPR-PReLU
Image is converted to gray-scale image	Image is converted to HSV array	Image is not disturbed and its contrast values of RGB values for relevant colors are extracted
Light colors of pixels are confused with other symptoms	Value of pixel does not signify the exact contrast of the required pixels	Required objects of the images are extracted exactly, since colors codes are applied
Ambiguity of identifying exudates	Color loss due to high intensity of value	Color is intact, and objects are selected
Two-value histogram is drawn and does not signify the existence of exudate symptoms	Full-color histogram is drawn, difficult to distinguish the objects with exudates	As only objects with exudate are developed, histogram signifies the intensity of exudates
Not possible to distinguish objects	Possible distinction of objects with much aberration	Objects are distinguished with very slight aberration

Total population: 89; samples: 35; average samples size: 40.

## Data Availability

The datasets used and/or analyzed during the current study are available from the corresponding author on reasonable request.
